# SNAREs: Could They be the Answer to an Energy Landscape Riddle in Exocytosis?

**DOI:** 10.1100/tsw.2010.137

**Published:** 2010-06-29

**Authors:** Wei Liu, Vladimir Parpura

**Affiliations:** Department of Neurobiology, Atomic Force Microscopy and Nanotechnology Laboratories, Center for Glial Biology in Medicine, Civitan International Research Center, and Evelyn F. McKnight Brain Institute, University of Alabama, Birmingham, AL, USA

**Keywords:** secretory vesicle fusion, SNARE, free energy, theoretical modeling, atomic force microscopy, surface force apparatus, isothermal titration calorimetry

## Abstract

During exocytosis, chemical transmitters stored in secretory vesicles can be released upon fusion of these intracellular organelles to the plasma membrane. In this process, SNARE proteins that form a ternary core complex play a central role. This complex could provide the means for generation/storage of energy necessary for driving the fusion of vesicular and plasma membranes. Recently, the amount of energy for (dis)assembly of the ternary complex has been measured using various experimental approaches, including atomic force microscopy, the surface force apparatus, and isothermal titration calorimetry. The obtained measurements are in good agreement with the calculated energy required for membrane fusion achieved by theoretical modeling approaches. Whether the energy expenditure to form the ternary SNARE complex can be utilized towards membrane fusion and/or docking/tethering of vesicles to the plasma membrane still remains one of the key contemporary issues in biophysics and neuroscience.

## INTRODUCTION

Fusion of secretory vesicles to the plasma membrane is thought to be mechanically driven by the interaction among the soluble *N*-ethylmaleimide-sensitive fusion protein (NSF) attachment protein receptors (SNAREs)[1]. In this process of exocytosis, the vesicular (v)-SNARE, synaptobrevin 2 (Sb2; also referred to as vesicle-associated membrane protein 2 [VAMP2]) forms a stable ternary *trans* complex when interacting with the binary *cis* complex that is preformed by two target (t)-SNAREs, the plasma membrane protein syntaxin (Sx) and the plasma membrane attached synaptosome-associated protein of 25 kDa (SNAP25) (Fig. 1). X-ray crystallography studies have revealed a bundled, coiled coils, appearance of the ternary complex, where Sx and Sb2 each provide one α coil/helix, while SNAP25 contributes two α coils. This four-helix bundle is held together by both an ionic interaction in the interior of the core at the so-called zero layer, and by hydrophobic bonds oriented almost perpendicular to the axis of the bundle and flanking the zero layer. The ionic layer serves as an alignment mark for coils within the ternary complex. It is thought that the ternary SNARE complex preponderantly assumes the parallel orientation under physiological conditions. In this arrangement, the N-termini of α coils, representing SNARE domains, of all contributing

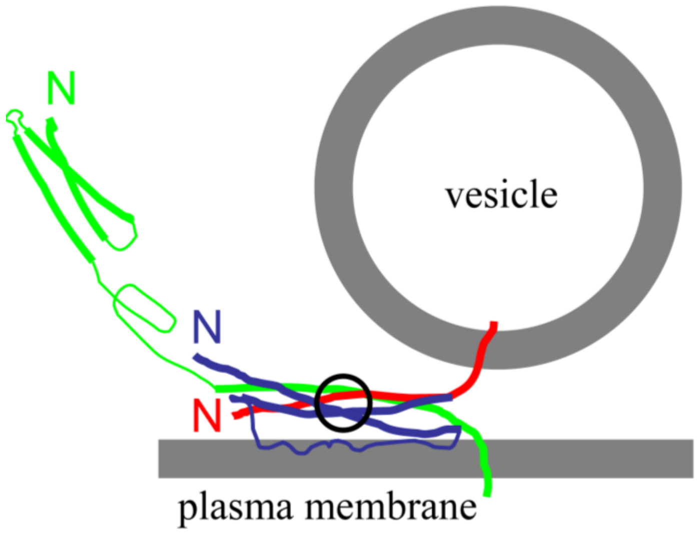

**FIGURE 1.** Exocytotic release of transmitter utilizes SNARE proteins. The ternary SNARE complex consists of Sb2 (red), located on the vesicular membrane; Sx (green) and SNAP25 (blue), both located on the plasma membrane. The proteins form the ionic zero layer (circle) in the mid section of the complex. Proteins are oriented in a parallel fashion. N, N-terminus. Drawings are not to scale.

partners are all positioned at one end[2]. In bulk biochemical experiments, the ternary Sb2-Sx-SNAP25 complex is thermally stable up to 90°C[3,4] as a consequence of stable interactions between coiled coils. It has been proposed that in SNARE-driven membrane fusions, the complex formation, which initiates at the N-termini end of coils and then proceeds towards the C-termini, progressively brings the vesicular and plasma membranes into contact, on which they eventually fuse[5,6]. Thus, the assembly of the SNARE complex could provide the mechanical force for docking/tethering of the vesicle to the plasma membrane and/or rearrangement of the membrane conformation to generate lipid bilayer curvature favorable for membrane fusion. The disassembly of the ternary SNARE complex requires the activity of the ATP-ase NSF and its cofactors, a process that displays a substantial “hysteresis” from the assembly of the complex[7]. Thus, the dynamics/energetics of SNARE interactions, lipid membrane contortion, and fusion are essential for our understanding of exocytosis[8,9].

## THEORETICAL MODELING OF MEMBRANE FUSION

There are two major approaches for theoretical modeling of membrane fusion: (1) a continuum approach based on treating membranes as macroscopic films and using classic mechanics of self-consistent mean field and elastic theory; and (2) molecular dynamic simulations using, for example, a coarse-grained method. Theoretical modeling of membrane fusion using a continuum approach suggests that it advances in three stages[10,11]: (1) stalk formation, (2) hemifusion, and (3) fusion with pore formation. It has been estimated that the formation of the stalk and hemifusion stages require 13 $k_BT$[10], while an energy barrier for full fusion is estimated to be about 46 $k_BT$[10]. Expansion of the fusion pore may require an estimated energy >50 $k_BT$[12]. Newer models of membrane fusion, mostly based on molecular dynamic simulations, indicate multiple pathways and lower energy requirements in this process[13,14,15] (Fig. 2). In addition to the above-outlined stalk-hemifusion-fusion pathway (Fig. 2, pathway I), an additional reaction pathway directly from stalk to fusion (Fig. 2, pathway II) can be observed[14]. Calculation of free energy indicates that stalk intermediate formation requires 6.4 $k_BT$, with an additional 5.5 $k_BT$ to reach the hemifused state, and then 4.3 $k_BT$ to the fused state (pathway I totaling 16.2 $k_BT$ from the starting configuration of two

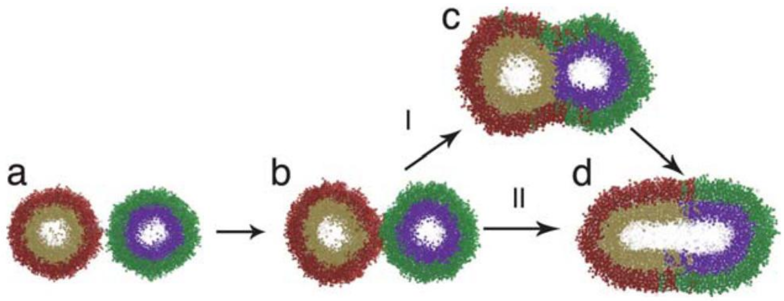

**FIGURE 2.** Model of membrane fusion. Two unfused lipid bilayers (a) form a stalk (b). This intermediate formation can advance to a hemifused state (pathway I; c) before the full fusion occurs (d). Alternatively, the hemifusion state can be by-passed (pathway II), achieving the fused state directly from the stalk state. Adapted from Kasson et al.[14].

unfused lipid bilayers to the fused state). By-passing the hemifusion state (pathway II), thus reaching the fused state directly from the stalk state, requires 10 $k_BT$, totaling 16.4 $k_BT$ for this pathway[14]. If SNAREs provide the driving force for vesicular and plasma membrane fusion, their (dis)assembly should generate sufficient force/energy that can be transferred to lipid bilayers, so that membranes can contort and undergo fusion. Recently, the energy requirements for the ternary SNARE complex (dis)assembly have been measured using various experimental approaches.

## EXPERIMENTAL MEASUREMENTS OF THE TERNARY SNARE COMPLEX ENERGETICS

Experiments attempting to quantify energetics of the ternary SNARE complex yielded measurements that are in good agreement with results provided by theoretical modeling of membrane fusion. Three different experimental approaches have been implemented thus far to measure the energy of the ternary SNARE complex (dis)assembly. The energy to take the ternary SNARE complex apart was obtained using the surface force apparatus (SFA) and atomic force microscopy (AFM)[16,17]. The energy to assemble the complex was measured by isothermal titration calorimetry (ITC)[18]. We expand on these approaches below.

### Single Molecule Dynamic Force Spectroscopy

AFM in a single molecule dynamic force spectroscopy (DFS) mode has recently emerged as a valuable technique for studying mechanical interactions between the proteins of the ternary SNARE complex ([17,19,20]; reviewed in [21,22,23]). In these studies, SNARE proteins were deposited onto the AFM tips and glass coverslips. Both surfaces were precoated with a thin nickel film (thickness ~150 nm) and after oxidation in air, $Ni^{2+}$ was used to attach six histidine-tagged recombinant proteins via steric coordination. Cytosolic domains of rat Sx1A amino acids (aa 1-266) and Sb2 (aa 1-94) were generated with six histidines at their C-termini, while the full-length rat SNAP25B was used. AFM tips functionalized with the binary Sx1A-SNAP25B complex were used to probe coverslips functionalized with Sb2 (Fig. 3A, inset). The coverslip mounted onto a piezoelectric tube was moved toward the tip. Due to the directional immobilization approach, the interaction between proteins on the tip/coverslip occurred in a physiologically more abundant parallel fashion ([24,25,26]; see discussion [19]), which initiated at the N-termini and progressed towards the C-termini. At the contact site with the coverslip, a binary Sx1A-SNAP25B complex attached to the tip bonded with the Sb2 deposited onto the coverslip to form a ternary Sb2-Sx1A-SNAP25B complex. Retracting the coverslip dissociated this complex, while rupture forces for

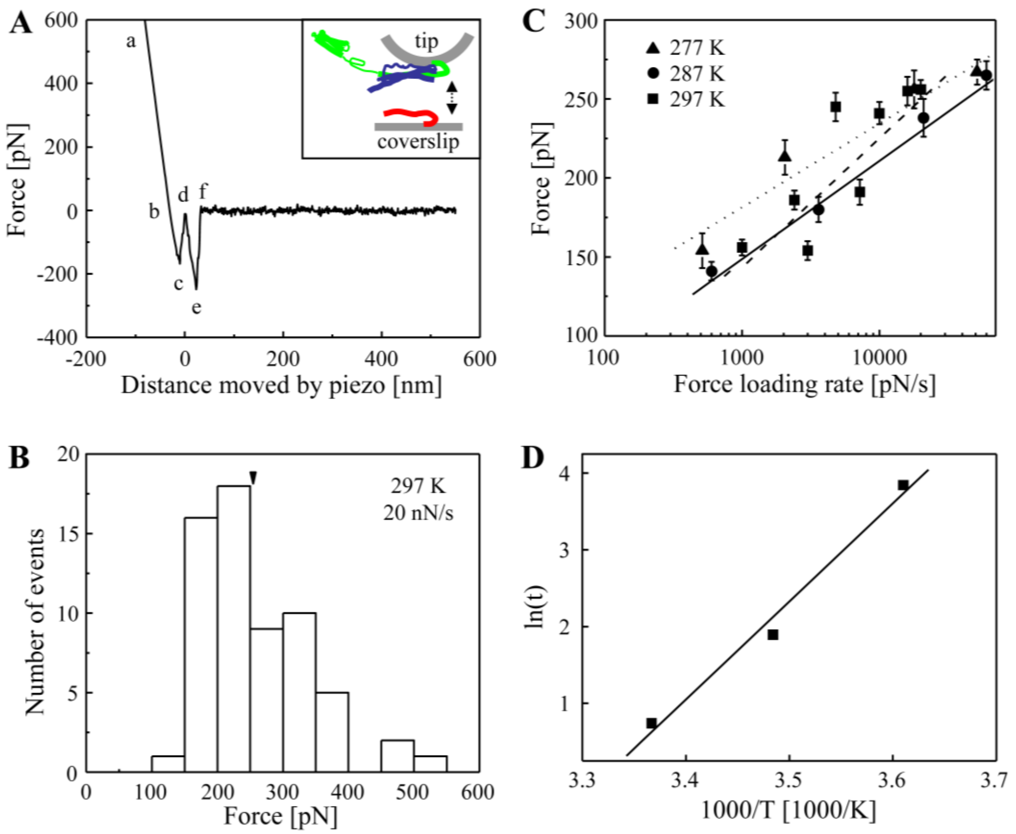

**FIGURE 3.** DFS approach for obtaining the disassembly energy of the ternary SNARE complex. (A) The inset (top right corner) represents a schematic of the experimental approach. The recombinant cytoplasmic domain of Sb2 (red) is attached at its C-terminus to the nickel-coated coverslip, allowing Sb2 interaction with the preformed binary complex containing SNAP25B (blue) bound to the cytoplasmic domain of Sx1A (green), and the latter being attached to the nickel-coated cantilever tip. These proteins are brought into contact by means of the piezoelectric element and then taken apart (double arrow). The graph shows the retraction part of a typical force-distance curve using an Sx1A + SNAP25B functionalized tip and an Sb2 functionalized coverslip. In the segments "*ab*" and "*bc*", the coverslip and the cantilever tip are still in contact. The Sb2-Sx1A-SNAP25B intermolecular "bond" starts to be extended at point "*d*", which represents the point of zero separation distance between the tip and coverslip. The increasing extension as the coverslip moves further away from the tip leads to increased application of the force on the intermolecular bond until it ruptures at point "*e*". The segment "*ef*" is then the measure of the force (ordinate) necessary to remove the interaction between the proteins of the ternary SNARE complex. (B) The distribution of the forces at rupture for Sb2-Sx1A-SNAP25B single complex intermolecular interactions obtained at 297 K (24°C) and the force loading rate of 20 nN/sec. The arrowhead represents the mean force at rupture. (C) The force values for dissociation of the SNARE proteins as a function of the force loading rate for three different temperatures of 297 K, 287 K (14°C), and 277 K (4°C) are shown as squares, circles, and triangles, respectively. Each point represents the mean ± sem. The force necessary to take the ternary complex apart increases exponentially with the increase in the loading rate. The corresponding best fits, shown as straight solid, dashed, and dotted lines, when extrapolated to F = 0 are used to obtain spontaneous dissociation times. (D) The natural logarithm of the spontaneous dissociation time (t) for the ternary Sb2-Sx1A-SNAP25B complex plotted as a function of the inverse temperature. The straight line indicates the best fit to the data, where the slope and the intercept represent ΔH and ΔS, respectively. Modified from Liu et al.[17]

this type of intermolecular interaction were measured (Fig. 3A). By repeating this cycle, a force histogram for dissociation of a single ternary SNARE complex can be generated (Fig. 3B). A typical average force of 256 pN for disassembly of the individual ternary SNARE complex at a pulling speed of 1.5 μm/sec (corresponds to 20 nN/sec of force loading rate) and temperature of 297 K (24ºC) was obtained. According to chemical reaction rate theory, the rupture force increases exponentially with the force loading rate (Fig. 3C). Extrapolating the force loading rate to zero force enabled estimation of dissociation rates, which correspond to the spontaneous lifetime[27]. For example, a spontaneous lifetime of the ternary SNARE complex was 2.1 sec[17] at 297 K (Fig. 3C). Based on the temperature-dependent chemical reaction rate theory, spontaneous lifetime (t) of a bond depends on the temperature (T) and the change in enthalpy (ΔH) according to the equation:

$$t = t_D * \exp(\Delta H / k_B T) \quad (1)$$

$$\text{where } t_D = t_a * \exp(-\Delta S / k_B) \quad (2)$$

and ΔS is the change in entropy, while $t_a$ is a time factor (derived from frequency factors of $6.2*10^{12}$ $sec^{-1}$ or $3.3*10^{9}$ $sec^{-1}$ for Eyring and Kramers models, respectively). When dissociation times were measured at three different temperatures, the interaction energy between the proteins of the ternary SNARE complex can be obtained from the Arrhenius plot containing the relationship between the spontaneous dissociation time and inverse temperature (Fig. 3D). It is important to realize that here the interaction energy actually is the adhesion energy between interacting protein molecules. In DFS, the change in adhesion energy corresponds to the ΔH of the intermolecular interactions according to ΔH = ΔG + TΔS, where ΔG is the change in free (Gibbs) energy. Thus, one can calculate ΔG after obtaining ΔH and ΔS from the fit to the data in the Arrhenius plot, where the slope represents the ΔH, while the intercept corresponds to the ΔS. Liu et al.[17] reported ΔH of 43 $k_BT$ and ΔG of 23–30 $k_BT$ for disassembly of the single ternary SNARE complex (Table 1).

**TABLE 1**
**Energetics of (Dis)assembly of the Ternary SNARE Complex**

| | | | ***ΔH*** | ***–TΔS*** | ***ΔG*** |
|---|---|---|---|---|---|
| **Disassembly** | **Surface 1** | **Surface 2** | | ***($k_BT$)*** | |
| AFM | $Sx1_{cd}$-SNAP25 | $Sb2_{cd}$ | 42.9 | –12.9*/–19.9** | 30.1*/23.0** |
| SFA | $Sx1_{cd}$-SNAP25 | $Sb2_{cd}$ | | | 35 |
| **Assembly** | **Cell** | **Syringe** | | | |
| ITC | ΔN complex | $Sb2_{cd}$ | –49.9 | 30.2 | –19.7 |
| | ΔN complex | $Sb2_{1-52}$ | –37.9 | 20.2 | –17.7 |

Modified from Liu and Parpura[9]; cd, cytosolic domain; * and ** denote Eyring and Kramers models, respectively.

## The Surface Force Apparatus

The SFA can directly measure the interaction force/energy between two facing surfaces as a function of their separation distances. It has been recently used to determine the free energy for dissociation of the ternary SNARE complex[16]. Lipid bilayers were deposited onto mica surfaces. The outer lipid layers (away from the mica surface) were functionalized by anchoring SNARE proteins to them. One surface/bilayer contained the cytoplasmic domain of mouse Sb2, while the other surface/bilayer was functionalized with the cytoplasmic domain of rat Sx1A. The attachment of the cytoplasmic domains of Sb2 and Sx1A was achieved by reacting their C-terminal single cysteine residues, introduced by recombination, to the head groups of maleimide-containing lipids of the outer lipid layer. The full-length mouse SNAP25 was precomplexed to Sx1A, forming the binary complex. Following functionalization, force measurements were carried out between two cross-cylindrical mica surfaces, containing lipids and SNAREs, as a function of separation distances (Fig. 4A). During the approach (Fig. 4B, Repulsion) of two bilayers containing v- and t-SNAREs, force was undetectable until their separation distance was

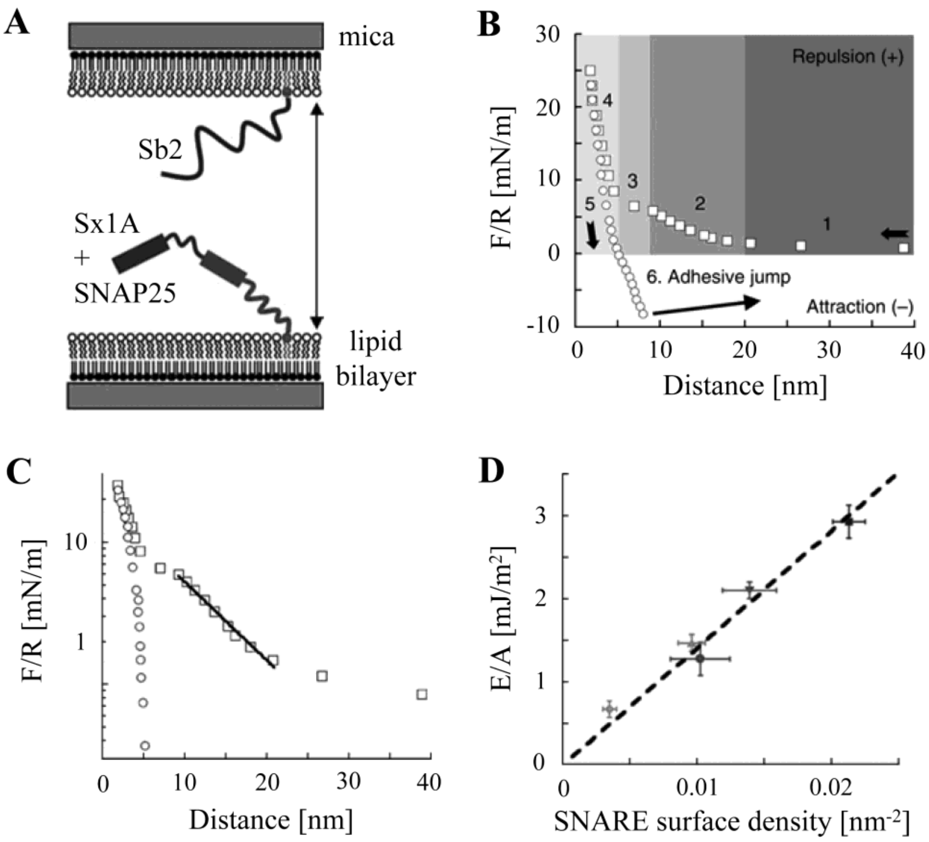

**FIGURE 4.** The SFA approach for obtaining the disassembly energy of the ternary SNARE complex. (A) Schematic of the experimental approach. The recombinant cytoplasmic domain of Sb2 is attached at its C-terminus to the outer layer of the lipid bilayer predeposited onto a mica surface. The cytoplasmic domain of Sx1A is attached using the same approach onto an apposing mica surface. SNAP25 is precomplexed to Sx1A so that these proteins form a binary SNARE complex. Two mica surfaces containing SNARE proteins anchored to the lipid bilayers are brought into contact and then taken apart (double arrow) by means of a leaf spring supporting one of the mica cylinders. Drawing is not to scale. (B) The graph shows the typical force-distance curve during the assembly (approach, horizontal arrow) and disassembly (downward arrow) of ternary Sb2-Sx1A-SNAP25 complexes. Numerated (1–5) and shaded areas indicate various segments of the experiment (see text for details). During the approach, there are repulsion (+) forces developed between proteins, while the adhesion between them causes attractive forces (–) during the retraction of mica surfaces. The ternary SNARE complexes are dismantled at a finite distance and force detected as an adhesive jump (point 6, long arrow) in the graph. The force (F) is normalized to the radius of the curvature (R) of the mica surfaces/cylinders. (C) Measurement of SNARE surface density. The measurements in area 2 of the approach, which report on long-range steric repulsions between SNAREs, can be used to deduce the SNARE surface density using a fit to the mushroom model (solid line). (D) The ternary SNARE complex free energy. The adhesion energy of SNAREs per unit area is plotted as a function of their surface density. Each symbol represents mean ± sem of measurements originating from independent pairs of mica surfaces. The dashed line represents the fit to the data. The slope of this line corresponds to the change of free energy due to disassembly of a single ternary SNARE complex. Modified from Li et al.[16].

reduced to 20 nm (Fig. 4B, area 1, horizontal arrow). As the surfaces were brought closer to each other, at distances separating them 8–20 nm, exponential repulsive forces were detected as a result of steric repulsions between SNARE proteins (Fig. 4B, area 2). This repulsion reached a plateau, indicating the occurrence of conformational changes between interacting SNARE proteins (Fig. 4B, area 3). As the approach continued, SNARE proteins at two surfaces were pushed against each other within short-range distances (~5 nm), resulting in their compression, which was detected as steep persistent exponential repulsion forces (Fig. 4B, area 4). The minimal limiting distance separating two cylinders of 2 nm was reached. This distance most likely reflects the thickness of the assembled ternary SNARE complexes, presumably in parallel orientation, since proteins were directionally immobilized. After the completion of an approach, the retraction part of the cycle followed (Fig. 4B, area 5, downward arrow). As the cylinders were retracted, adhesion/attraction forces that developed between SNAREs were detected (Fig. 4B, Attraction). This was especially evident at distances over 5 nm. As the separation distance between cylinders increased, pulling forces were applied to stretch SNARE bonding. The rupture of bonds between the proteins of the ternary SNARE complexes occurred at ~8 nm, an event displayed as an adhesive jump in the graph (Fig. 4B, point 6). Because of crossed-cylinder geometry used in experiments, (dis)assembly of multiple SNARE complexes occurred simultaneously at various distances. Consequently, the force measured at any given distance is the sum of forces occurring between two surfaces at distances defined by the radial curvature (R) of mica cylinders. At equilibrium, surface force (F) is related by the Derjaguin approximation to the interaction free energy (E) according to the equation:

$$E = F/2\pi R \tag{3}$$

Thus, the force measurements obtained using the SFA represent total integrated force over the entire area of contact between two mica surfaces, which contain many SNARE complexes. Consequently, the free energy for disassembly of a single ternary SNARE complex can be calculated if the number of interacting complexes per unit area can be determined. The average density of one protein per 121 $nm^2$ was deduced by modeling long-range steric repulsions during the approach in the experiments utilizing the SFA (Fig. 4C) and using the "mushroom" theory developed for low polymer density. From the data of pull-off forces at adhesion jump, the plot of energy per unit area vs. the surface density of SNAREs was generated (Fig. 4D). By fitting data within this plot, a linear relationship was obtained. The slope of the fit represents the change in energy between bound and unbound states of a single ternary SNARE complex. The obtained change in energy of 35 $k_BT$ corresponds to the free, presumably Gibbs, energy required to dissociate the single ternary SNARE complex[16] (Table 1). Taken together, the measurements of free energy necessary to disassemble a single ternary SNARE complex obtained by DFS and the SFA are in reasonably good agreement. Both of these approaches, however, could not be used to measure the energetics of the assembly, which has been measured by calorimetry[18].

## Isothermal Calorimetry

ITC has been used to measure directly the thermodynamic properties of the individual steps in ternary SNARE complex assembly[18]. Various SNARE proteins in solution were put in a thermally insulated cell and syringe, and then were mixed by injection from the syringe to the cell. During mixing, the interaction of molecules occurred, while the heat changes ($\Delta Q$) were measured. At isobaric conditions, $\Delta Q$ is equal to $\Delta H$ of the reaction. Additionally, when using a titration approach, the stoichiometry and $\Delta S$ were also obtained, so that $\Delta G$ could be calculated. Since ITC experiments are executed in solution lacking directional immobilization of the proteins, there are two issues that need to be resolved to obtain meaningful data. (1) The assembly carried out in solution may result in a mixture of nonsequentially formed complexes[9] in antiparallel and parallel alignment configurations[28]. These configurations are a result of interactions between the SNARE domains of Sx1A and Sb2, while SNAP25 remains parallel to Sx1 at all times[28]. (2) Isolating Sb2 binding to the binary Sx-SNAP25 complex in solution is difficult since the complex exists in equilibrium between 1:1 and 2:1 stoichiometry, the latter being a "dead-end species"[29] as it does not bind Sb2[5]. To assure the sequential ternary SNARE complex formation of the physiological, parallel binding of Sb2 to a preformed Sx1-SNAP25 binary complex with 1:1 stoichiometry, Wiederhold and Fasshauer[18] chose a clever experimental design for the ITC experiments. They used a partially assembled complex, the so-called $\Delta N$ complex[5], in which 1:1 Sx-SNAP25 binary complex can be stabilized by addition of C-terminal fragments of the Sb2 SNARE domain (aa 49-96), and then purified (Fig. 5A,B). All recombinant proteins were derived from rat cDNA sequences; Sx1A lacking the $H_{abc}$ domain and the linker region, but having the SNARE domain (aa 180-

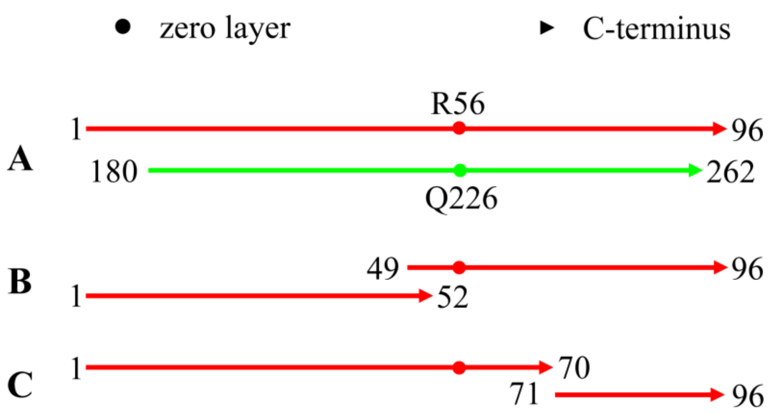

**FIGURE 5.** Linear alignment of Sx1A (green) and Sb2 (red) fragments used in ITC. SNAP25A was left out from the drawing for simplicity. All proteins are shown in the parallel orientation with their C-termini indicated by arrowheads. (A) The ternary complex aligns at the zero layer in which Arg56 and Gln226 residues (shown by dots) of Sb2 and Sx1A SNARE domains ($Sb2_{1-96}$ and $Sx1A_{180-262}$), respectively, combined with two Gln from SNAP25A form the layer. (B) In the ΔN complex, a binary Sx1A-SNAP25A complex is stabilized by the $Sb2_{49-96}$ fragment, which can additionally bind $Sb2_{1-52}$ to form a complex resembling the ternary SNARE complex. When the entire cytosolic domain of $Sb2_{1-96}$ binds to the ΔN complex, it displaces $Sb2_{49-96}$. (C) Alternatively, the Sx1A-SNAP25A complex can be stabilized using $Sb2_{1-70}$ in lieu of $Sb2_{49-96}$, which can bind a complementary C-terminal $Sb2_{71-96}$ fragment.

262), and the cysteine-free SNAP25A (aa 1-206) were used. The stabilizing fragment $Sb2_{49-96}$ contains Arg56, which contributes to the formation of the ionic zero layer of the ternary SNARE complex. Thus, the ΔN complex ionic layer is flanked with two N-terminal and eight C-terminal hydrophobic layers (for detailed structure see Sutton et al.[2]). Due to this asymmetry of the Sb2 stabilizing fragment, we shall make an assumption here that it mainly engages in parallel orientation binding within the ΔN complex as it aligns at the zero layer (Fig. 5B). The ΔN complex was titrated by injection of the entire cytosolic domain of $Sb2_{1-96,}$ which binds to the complex and displaces $Sb2_{49-96}$, yielding ΔH of –49.9 $k_BT$ and ΔG of –19.7 $k_BT$ (Table 1). One concern with this approach is what proportion of the observed energy is related to the binding of the cytosolic domain $Sb2_{1-96}$ to the complex as opposed to the displacement of the $Sb2_{49-96}$ fragment. To address this issue, two additional truncated Sb2 forms were used. Hence, the ΔN complex was titrated with a shorter N-terminal fragment of Sb2 SNARE domain $Sb2_{1-52}$. Although $Sb2_{1-52}$ has four overlapping amino acids with the C-terminal $Sb2_{49-96}$ fragment, it binds to the ΔN complex without displacing the $Sb2_{49-96}$ fragment. Thus, two overlapping Sb2 fragments bind at the same time to an Sx-SNAP25 complex, forming a complex resembling the ternary SNARE complex (Fig. 5B). Corresponding ΔH (–37.9 $k_BT$) and ΔG (–17.7 $k_BT$) during this event were similar to those obtained when a ΔN complex was titrated by injection of $Sb2_{1-96}$ (Table 1). This indicated that binding of the N-terminal part of Sb2 contributes to most of ΔH and ΔG in Sb2 binding to the binary Sx-SNAP25 complex. Consequently, C-terminal Sb2 binding to the complex should play a lesser role in energetics of the ternary SNARE complex. This hypothesis was further tested by ITC experiments in which the preformed ΔN complex contained $Sb2_{1-70}$ in lieu of $Sb2_{49-96}$ (Fig. 5C). Binding of a complementary C-terminal $Sb2_{71-96}$ fragment to the complex displayed much reduced ΔH (–10 $k_BT$) and ΔG (–13.4 $k_BT$) when compared to other measurements obtained using the ΔN complex comprised of fragments as outlined above. Taken together, it appears that binding of Sb2 to a preformed Sx-SNAP25 heterodimer generates ΔG of about –20 $k_BT$ as measured by ITC, a value that is in good agreement with that obtained for the disassembly of the ternary SNARE complex using AFM (Table 1). Since SNARE assembly and disassembly display substantial hysteresis[7], any comparisons between energy measurements obtained thus far should be done with great caution. In analogy of SNARE (dis)assembly to the action of muscles[30], one should not draw inferences about flexing power based on extension, and vice versa. However, the experiments using the SFA, AFM, and ITC were done in isolation of other interacting proteins and complex lipids otherwise present in intracellular milieu; such isolated nature of experiments leads to more relevant comparisons. Of course, the fact that measurements were obtained using different techniques that have various (dis)advantages (see discussion in Parpura and Mohideen[22]), and slight variations in protein sequences and truncations used, should also be taken into contemplation.

## CONCLUDING REMARKS

The experimentally obtained free energy of 20–35 $k_BT$ generated during the (dis)assembly of a single ternary SNARE complex[16,18,23] is similar to the amount of energy needed to cause hemi- or full fusion of two lipid bilayers based on theoretical modeling[10,11,13,14,15,31]. It appears that a minimal number (single or a few) of ternary SNARE complexes could generate a sufficient amount of energy to cause vesicular and plasma membrane fusion. Indeed, this is in agreement with recent experiments using liposomes, where one ternary SNARE complex was sufficient for membrane fusion[32]. However, how energy is transferred from the bundling between the SNARE domains of the proteins within the core complex into lipid bilayers that ultimately need to reshape in membrane mergers is still unclear. Two recent studies suggest that the linker region between the SNARE domains representing the core of the complex and the transmembrane domains may play a role in this process[33,34]. Hence, X-ray structural findings indicate that assembly of the SNARE complex may continuously proceed beyond the already known core ternary SNARE complex to encompass the C-terminal linker regions where additional helical bundling is stabilized by side-chain interactions[34]. Additionally, nuclear magnetic resonance data of the full-length lipid-bound Sb2 revealed its dynamic structure, most notably a transient helix that extends C-terminally from the SNARE domain into the transmembrane domain[33]. These findings suggest that the SNARE complex assembly could be coupled to the act of membrane fusion, whereby SNAREs may operate as nanomachines, directly providing a driving force to overcome the energy barrier of membrane fusion. This scenario could play out in “crash fusion”, where vesicles fuse to the plasma membrane without becoming first stably docked/primed (reviewed in [35]). When SNARE complex formation would take place in the ready-releasable pool of vesicles[36], the energy released from the assembly of the minimal number of ternary SNARE complexes could be consumed during the docking/priming process. Consequently, there should be an additional source of mechanical force/energy to drive fusion of the ready-releasable pool of vesicles. Perhaps this can be achieved by the chronological recruitment of the additional assembly of ternary SNARE complexes over their “quota” needed for docking of vesicles. It should be noted that only one ternary SNARE complex should be sufficient for subsequent membrane fusion since multiple complexes did not act synergistically in this process[32]. Alternatively and/or additionally, the interactions of the ternary SNARE complex with various lipids, e.g., sphingosine[37], and ancillary proteins, such as synaptotagmins and SM (Sec1/Munc18-like) proteins[38], could provide a needed energetic boost. Another intriguing issue is whether the isoform composition of the ternary SNARE complex, most notably SNAP25 vs. SNAP23, which display quite different thermal stability[3,4], would influence the energetics of the complex. Clearly, additional studies in regard to ancillary molecules to and isoform composition of the ternary SNARE complex would provide further clues necessary to answer the energy landscape riddle in exocytosis.

## ACKNOWLEDGMENTS

We thank Randy F. Stout, Jr. for his comments on previous versions of this mini-review. The authors’ work is supported by grants from the National Institute of Mental Health (R01 MH 069791 to VP) and the National Science Foundation (CBET 0943343 to VP).
